# The Cervical Microbiome over 7 Years and a Comparison of Methodologies for Its Characterization

**DOI:** 10.1371/journal.pone.0040425

**Published:** 2012-07-09

**Authors:** Benjamin C. Smith, Thomas McAndrew, Zigui Chen, Ariana Harari, David M. Barris, Shankar Viswanathan, Ana Cecilia Rodriguez, Phillip Castle, Rolando Herrero, Mark Schiffman, Robert D. Burk

**Affiliations:** 1 Department of Pediatrics, Albert Einstein College of Medicine, Yeshiva University, Bronx, New York, United States of America; 2 Department of Obstetrics, Gynecology and Women’s Health, Albert Einstein College of Medicine, Yeshiva University, Bronx, New York, United States of America; 3 Department of Microbiology and Immunology, Albert Einstein College of Medicine, Yeshiva University, Bronx, New York, United States of America; 4 Department of Epidemiology and Population Health, Albert Einstein College of Medicine, Yeshiva University, Bronx, New York, United States of America; 5 Proyecto Epidemiológico Guanacaste, Fundación INCIENSA, San José, Costa Rica; 6 American Society of Clinical Pathology (ASCP) Institute, Washington, D.C., United States of America; 7 Division of Cancer Epidemiology and Genetics, National Cancer Institute, Bethesda, Maryland, United States of America; Columbia University, United States of America

## Abstract

**Background:**

The rapidly expanding field of microbiome studies offers investigators a large choice of methods for each step in the process of determining the microorganisms in a sample. The human cervicovaginal microbiome affects female reproductive health, susceptibility to and natural history of many sexually transmitted infections, including human papillomavirus (HPV). At present, long-term behavior of the cervical microbiome in early sexual life is poorly understood.

**Methods:**

The V6 and V6–V9 regions of the 16S ribosomal RNA gene were amplified from DNA isolated from exfoliated cervical cells. Specimens from 10 women participating in the Natural History Study of HPV in Guanacaste, Costa Rica were sampled successively over a period of 5–7 years. We sequenced amplicons using 3 different platforms (Sanger, Roche 454, and Illumina HiSeq 2000) and analyzed sequences using pipelines based on 3 different classification algorithms (usearch, RDP Classifier, and pplacer).

**Results:**

Usearch and pplacer provided consistent microbiome classifications for all sequencing methods, whereas RDP Classifier deviated significantly when characterizing Illumina reads. Comparing across sequencing platforms indicated 7%–41% of the reads were reclassified, while comparing across software pipelines reclassified up to 32% of the reads. Variability in classification was shown not to be due to a difference in read lengths. Six cervical microbiome community types were observed and are characterized by a predominance of either *G. vaginalis* or *Lactobacillus* spp. Over the 5–7 year period, subjects displayed fluctuation between community types. A PERMANOVA analysis on pairwise Kantorovich-Rubinstein distances between the microbiota of all samples yielded an *F*-test ratio of 2.86 (p<0.01), indicating a significant difference comparing within and between subjects’ microbiota.

**Conclusions:**

Amplification and sequencing methods affected the characterization of the microbiome more than classification algorithms. Pplacer and usearch performed consistently with all sequencing methods. The analyses identified 6 community types consistent with those previously reported. The long-term behavior of the cervical microbiome indicated that fluctuations were subject dependent.

## Introduction

The cervicovaginal microbiome plays an important role in female reproductive health, affecting rates of preterm-birth and neonate mortality; prevalence, susceptibility to and transmissibility of STD’s (including HIV); and other important clinical conditions [1], [2], [3]. Moreover, recent studies indicate bacterial vaginosis, cervical inflammation and vaginal pH play a role in the susceptibility to and natural history of cervical HPV infection and the development of cervical intraepithelial neoplasia [4], [5], [6]. It has been suggested [7] that a possible route of bacterial colonization of intrauterine infections is through the cervix, which is typically considered a physical barrier aiding the maintenance of uterine sterility. Recent findings indicate a complex cervicovaginal microbe ecology that can be broadly characterized as a set of 5 categorical community types [8], [9]. Furthermore, differences in the distribution of these microbiome community types have been observed amongst women of different races [8], [10].

Next-Gen Sequencing (NGS) allows large numbers of molecules from single or multiple samples to be sequenced in a single run. This dramatically expands the horizons of microbiology, as it represents a departure from dependency on culture-based methods and low-throughput cloning and sequencing for identifying microorganisms. Two NGS technologies currently dominate the field. In one run, the Roche 454 GS-FLX system (hereafter referred to as “454”) can produce up to 1 million high-quality reads, the majority of which are ∼500 bp in length. The Illumina HiSeq2000 platform (hereafter referred to as “Illumina”) can produce up to approximately 450 million reads of high-quality sequence, with read lengths up to 120 bp. The maximum read length and number of molecules sequenced continue to grow as these and other technologies evolve [11].

The advent of NGS has allowed characterization of microbial life inhabiting specific ecological niches on an unprecedented scale [12]. This is enabled by amplification and parallel sequencing of fragments of genes that are highly conserved amongst microorganisms. The most commonly targeted gene, to date, is the 16S ribosomal RNA subunit gene, present in all known bacteria and Achaea. Other highly conserved genes with potential for characterizing communities of organisms in a sample include RecA and RpoB [13]. Highly conserved regions of the 16S rRNA gene, which facilitate PCR amplification, flank highly variable regions (V1–V9) that allow phylogenetic and taxonomic identification. Due to the length restrictions of NGS, any individual read only contains the sequence of a 16S rRNA gene fragment, thus choices as to which region will be targeted must be made during experimental design.

A caveat to performing taxonomic classification on short fragments of DNA arises from the variable taxonomic level to which any given sequence may be assigned. Fragments of the same size, from the same relative position in a conserved gene, but from different organisms, often contain different amounts of sequence variability that influence the classification to different taxonomic levels. For example, one fragment may contain only enough information to be identified to its family, whereas another otherwise similar fragment from a different organism may be identifiable below the species level. The latter occurs when the region in question is more diverse amongst closely related members of its genus. Each hypervariable region of the 16S genome spans approximately 100 bp, thus longer read lengths would be expected to provide more information for discriminating amongst the lower taxonomic levels by allowing several hypervariable regions to be sequenced at once [14].

A further caveat arises from having to accurately assign large numbers of reads to their originating organisms within a reasonable amount of computational time. There is no algorithm that can do this with complete accuracy, since this depends to a large extent on the quality of multiple sequence alignments, a notoriously hard problem. Numerous software algorithms exist for assigning bacterial taxa to NGS reads, however, comparisons of the performance of the most widely used and/or promising of these tools for reads generated by different sequencing technologies are scarce. Furthermore, the corpus of characterized 16S sequences isolated from existing microbes is incomplete, albeit rapidly growing [15].

**Figure 1:**
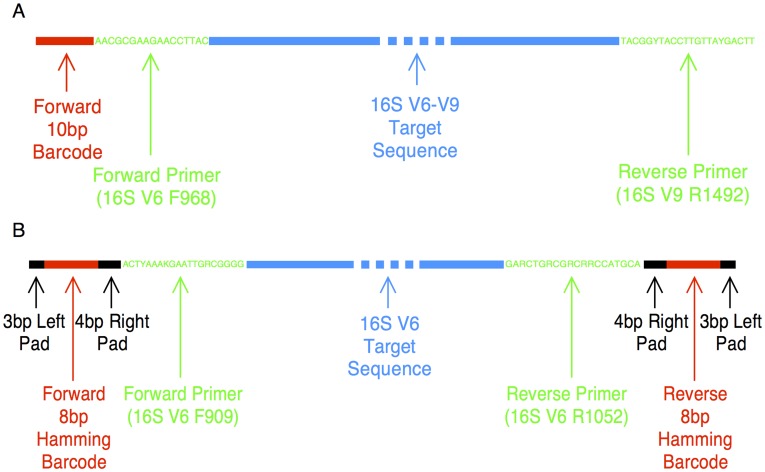
Primer design. Panel A shows the primer design for amplifying the V6–V9 16S rRNA gene region analyzed by cloning and Sanger sequencing and 454 pyrosequencing. Regular (non-encoded) 10 bp barcodes were added to the 5′end of the forward PCR primer. Panel B shows the primers used to amplify the 16S V6 region, analyzed by Illumina sequencing. Hamming barcodes (8 bp in length) [32] and padding sequences were introduced to the 5′ ends of the forward and reverse PCR primers, different for each strand, so that reads from each strand could be distinguished. Note: the reverse primer sequences shown are the actual oligonucleotide sequences used in PCR amplification (i.e., the reverse complement of the 5′–3′ target DNA sequence).

Here, we provide comparisons of the bacterial community compositions reported by three different sequencing technologies in combination with three different software analysis pipelines operating on amplicons of the V6 (143 bp) and V6–V9 (524 bp) regions of the 16S rRNA subunit gene (see Figure 1 for primer design), using a fixed database constructed for the cervicovaginal microbiome. Samples were obtained from a large, population-based cohort in Guanacaste, Costa Rica designed to study the natural history of human papillomavirus (HPV) and cervical neoplasia [16], [17]. In addition to the assessment of methodological variables, we performed an evaluation of the cervical microbiome and its stability in a subset of women sampled approximately annually over a 5–7 year period.

## Results

### Community Compositions by different Methods

**Figure 2:**
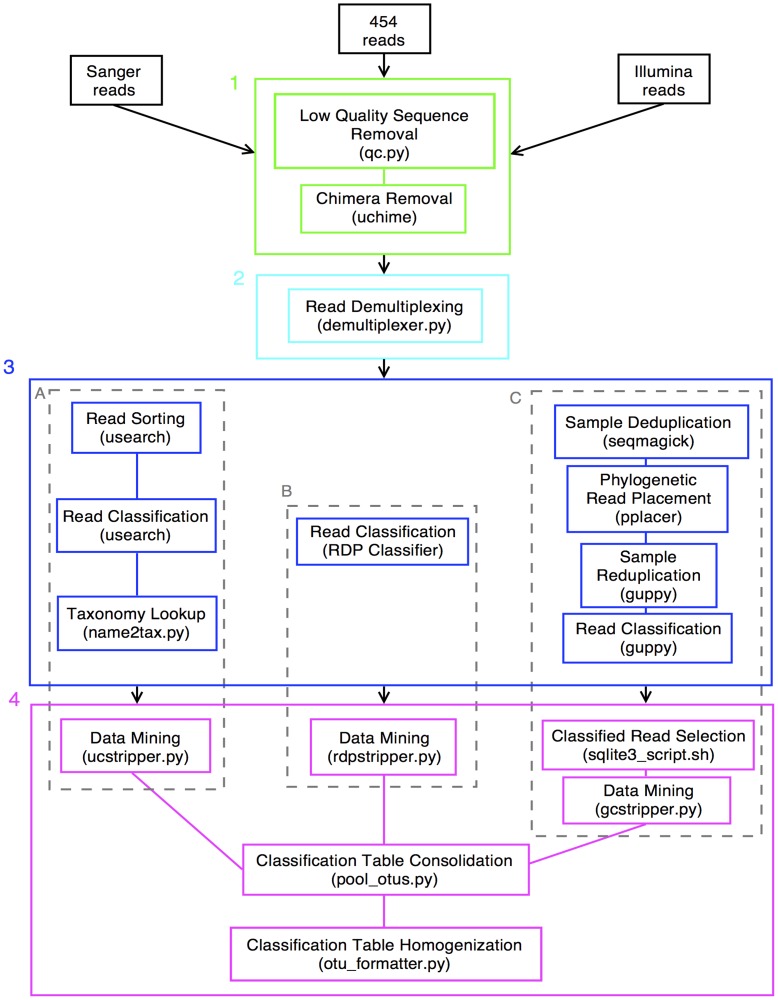
Flowchart of sequencing technologies, methodological pipelines and associated software. Sequencing files in FASTQ or FASTA, and QUAL formats underwent the following steps shown in the indicated panels: (panel 1) Quality filtering, where short and low quality reads were discarded and chimeric sequences were detected and removed; (panel 2) Read demultiplexing was performed where reads were assigned names according to the clinical sample from which they originated based on each unique barcode; (panel 3) Read identification was performed using (subpanel A) usearch, (subpanel B) RDP Classifier, and (subpanel C) pplacer. For usearch and pplacer, classification involved multiple processing steps and format modifications (panel 4) to allow for direct comparison between methodological configurations. The data standardizing scripts yielded tables containing the counts for each detected genus (rows) and clinical sample (columns). Some taxa appeared multiple times in the initial tables, therefore the counts for these taxa were pooled. Filtering was also applied to discard any counts that constituted <1% of the total sample composition. Taxa that were empty of counts across all samples after this low-pass filtering were discarded. Finally, to allow direct comparison, all nine classification-tables were formatted such that the numbers of rows and columns in each table were equal and contained a union of all taxa and samples.

Nine methodological pipelines for microbiome characterization were compared; a flowchart of the experiment is shown in Figure 2. Clinical samples were initially analyzed using Sanger sequencing of a mean of 47 (SD = 10) clonal isolates of bacterial 16S V6–V9 amplicons (the “universal” primer sequences can be found in Fig. 1A). This provided a tractable set of sequences obtained from readily available molecular biology methods, against which to compare massive amounts of sequencing data from emerging, complicated NGS protocols. To date, 454 sequencing has been the most frequently used platform for microbiome analyses, primarily due to its longer read lengths. However, its high cost per run is a limiting factor for many laboratories. We sequenced a mean of 4380 (SD = 3650) V6–V9 amplicons (see Fig. 1A for primers) for each clinical sample using the 454 system. Illumina platforms provide shorter reads, but deeper coverage and at significantly lower cost. Using Illumina, we obtained a mean of 29400 (SD = 13340) reads for each V6 amplicon (see Fig.1B for primers).

Three primary software tools were chosen as a basis for sequence analyses; assigning each read to its originating genus or species of microbe. The tools were selected based on their popularity as inferred from studies presented at the Human Microbiome Research Conference (St. Louis, MI, USA, in August of 2010), usearch [18] and RDP classifier [14], and an emerging software package, pplacer [19], that employs a statistically rigorous, phylogenetically oriented approach that may provide important analytical advantages. Numerous manipulations between data and database formats were required to allow precise comparison of sequences from the 3 molecular methods, classified with the 3 software pipelines. These required additional scripts, developed in-house at Einstein (see Methods section).

**Figure 3:**
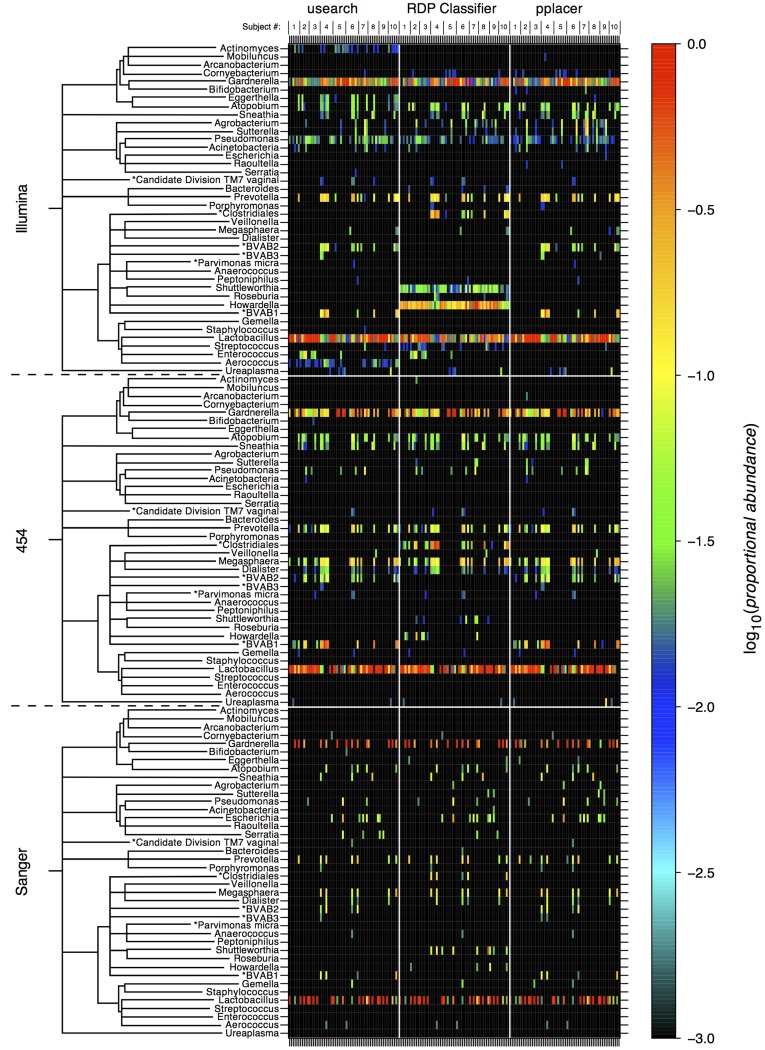
Community compositions of cervical samples at the genus level as determined by 9 different methodological configurations. Heat-maps show the log_10_ (*proportional abundance*) of each bacterial genus detected in each clinical sample for each methodological combination. In the 3×3 grid of heat-maps, the sequencing method is indicated at the far left and the classification software is indicated at the top of the panels. The cladograms to the left of the genus names indicate the approximate evolutionary relationships between genera.

The bacterial community composition of every sample determined by each of the methodological configurations is shown in 9 similarly organized panels of heat-maps (Figure 3). The proportional amount of each genus detected is represented as a colored cell, with red indicating 100% abundance within a sample and black indicating ≤0.1%. A cladogram to the left of the heat-maps was adapted from a maximum likelihood phylogenetic tree based on the complete 16S genes of the detected genera and displays the approximate evolutionary relationship between each bacterial genus detected within the set of samples. Methodologies were evaluated at fixed taxonomic levels, as opposed to employing a floating “operational taxonomic unit” (OTU) classification, to facilitate direct comparison and visualization. The genus level was chosen since this is the lowest common taxonomic assignment level amongst the classification software we employed (i.e., RDP classifier cannot classify below the genus level). Clinical samples in Fig. 3 are arranged chronologically from left to right for each subject. The figure shows that two genera dominate the cervical microbiota of these subjects across all methodologies: *Lactobacillus* and *Gardnerella*, in agreement with previous studies of female reproductive tract microbiota [8], [9], [20], [21]. In addition, depending on the sequencing and classification method, relatively high proportions of *Prevotella, Megasphaera*, *BVAB1/Clostridiales* and *Howardella* were observed (Fig. 3). It should be noted that for some sequences in the 16S database, the genus to which the isolates belonged had not been characterized. Nevertheless, there existed reads that were confidently assigned identities from these sequences at the genus level or lower. Often the species name was taken as the genus-level identifier, for example, *BVAB1, 2* or *3* are species belonging to the *Clostridiales* order, where neither family- nor genus-level information was available. Usearch and pplacer classified a proportion of reads as either *BVAB1, 2* or *3* and these were thus assigned both as the species and genus name, in the absence of an appropriate genus-level identifier. However, RDP Classifier was unable to distinguish between these three species and could not assign them to separate genus-level identifiers (note their absence in the RDP Classifier panels, middle panels in Fig. 3), so they were classified as originating from a genus within the *Clostridiales* order (note their presence here for the RDP Classifier panels in Fig. 3). In all such cases, the named consensus lineages are annotated (with an *) to indicate that the order or species name corresponds to an unknown genus. A forthcoming release of the vaginal microbiome reference package will offer improved classification of the BVAB species (Frederick Matsen, personal communication).

**Figure 4:**
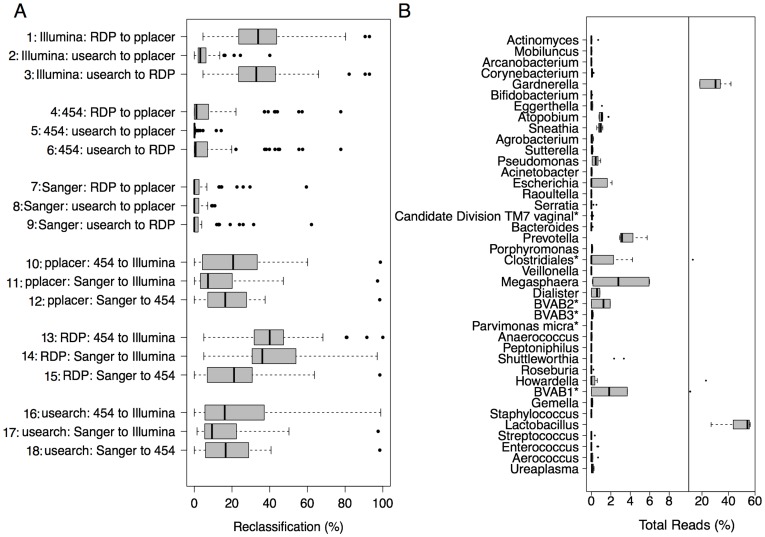
Microbiome reclassification by different methodological configurations. Panel A shows a boxplot of the percentage of total reads reclassified as different genera for all samples, for each pairwise comparison between methodologies. Outliers beyond the interquartile range are shown as points. Panel B shows the percentage of total classified reads assigned to each genus. The distributions reflected by the boxplots indicate the variability of the classification percentages between methodological configurations.

To quantitate the changes that resulted from either the same data being analyzed by different software or data from different sequencing platforms analyzed by the same software, we produced boxplots of the proportional reclassification that occurs between methods (Fig. 4A). To assess which of the genera showed the largest variability between methods, we show the distributions of the total proportions of reads assigned to each genus by each methodological configuration (Fig. 4B).

Transitions between classification algorithms for each sequencing platform (Fig. 4A, rows 1–9) showed lower median reclassification of reads than the transitions between sequencing platforms for each classification algorithm (Fig. 4A, rows 10–18). Illumina sequencing in combination with the RDP algorithm produced the most strikingly different community compositions compared with the other methodological configurations –32%–41% of reads were reclassified (Fig. 3, top-center panel and Fig. 4A, rows 1, 3, 13 & 14). The RDP Classifier in conjunction with 454 or Sanger sequencing produced classifications consistent with those of the other software methods (Fig. 4A, rows 4, 6, 7 & 9), but was least consistent when compared across sequencing methods (Fig. 4A, rows 13, 14 & 15 vs. 10, 11, 12, 16, 17 & 18). Pplacer and usearch gave consistent classifications for each pairwise comparison between sequencing methods (Fig. 4A, rows 10 vs. 16, 11 vs. 17 & 12 vs. 18), with the Sanger to Illumina comparisons showing the lowest median reclassification (Fig. 4A, rows 11 & 17).

**Figure 5:**
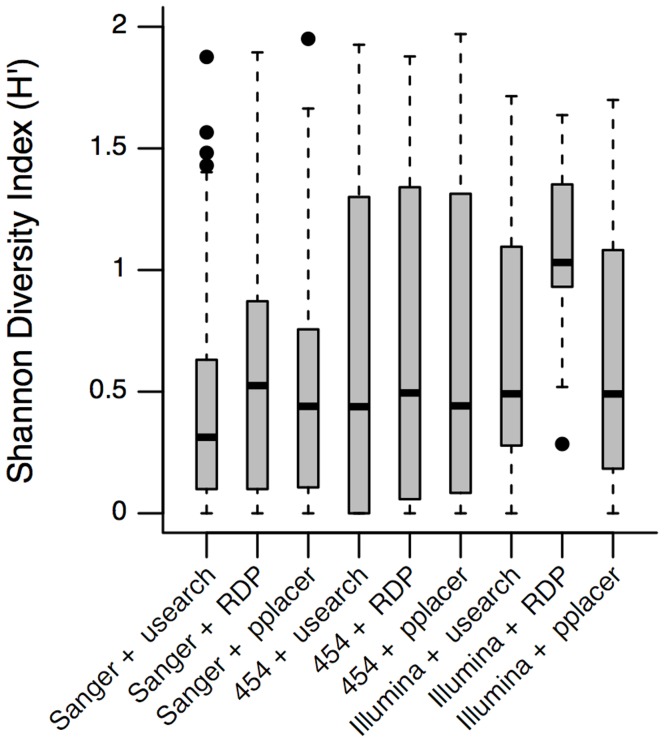
A comparison of the Shannon diversity indices for each methodology. The Shannon diversity index (*H’*) was calculated based on the genus-level classification tables produced by each combination of sequencing method and software pipeline. The boxplots show the distribution of *H’* values across all samples. For a given sequencing method, the Shannon diversity index appears consistent across classification software, except for the Illumina and RDP Classifier combination, where a large increase in apparent diversity occurs.

The large reclassification that occurred when using RDP Classifier to catalog Illumina reads accompanied a large increase in diversity, as measured by the Shannon diversity index (Fig. 5). All other methodological configurations produced similar median diversities, suggesting that the anomalously large value produced by RDP Classifier with Illumina reads occurred as a result of its purported inaccuracy for sequences <250 bp in length [22], [23]. Furthermore, although only small numbers of reads were generated in the cloning and Sanger sequencing approach, it nevertheless appeared capable of capturing most of the microbial diversity present in the samples. The implication of this for NGS is that it should be possible to sequence many thousands of cervical samples in a single run, without falling below the minimum necessary depth to capture the majority of the diversity.

Variation in the proportion of reads assigned to each genus across all 9 methodologies can be seen in Figure 4B. The largest overall uncertainty occurred for *Lactobacillus* and *Gardnerella*, the two most abundant genera. *Megasphaera*, although a small component of the overall microbiome, shows a large degree of variation relative to its abundance. *Prevotella*, by contrast, shows a similar abundance but less than half the variability of *Megasphaera*. Most of the variability in *BVAB1, 2* and *Clostridiales* results from missing information in the database introducing classification discrepancies between RDP Classifier vs. usearch and pplacer, as discussed earlier.

### Truncating 454 Reads does not alter the Assigned Microbiome

Sequence data from the Roche 454, spanning the V6–V9 regions, was truncated to cover just the V6 region (equivalent to the region determined by Illumina sequencing) and truncated reads were phylogenetically assigned by pplacer. The normalized Kantorovich-Rubinstein (KR) distances (Z*_p_*) between the placement distributions of truncated 454 reads, the full-length 454 reads, and the Illumina reads (Table 1) with (weighting parameter) *p* = 1 [24] were calculated. Classifications using truncated and full-length 454 reads were nearly identical (*Z_p_* = 1.8×10^−4^), whereas those between truncated or full-length 454 reads and Illumina reads had distances two orders of magnitude greater (*Z_p_* = 1.6×10^−2^). This result supports the notion that PCR bias due to differential primer specificity is likely to be responsible for classification differences between sequencing methods, rather than a difference in information content between longer and shorter reads.

**Table 1:** Comparing the overall Kantorovich-Rubinstein distance between truncated and full-length sequencing data.

*Sample 1*	*Sample 2*	*KR-Distance*
454	Truncated 454	1.8×10^−4^
454	Illumina	1.6×10^−2^
Illumina	Truncated 454	1.5×10^−2^

Quality controlled 454 sequencing data longer than 120 bp was truncated and processed using the pplacer pipeline (Fig. 2). Merged placements from the truncated data for all 60 samples were compared to the same full-length (454 and Illumina) data by calculating the tree-length normalized KR distances between all pair-wise combinations of the three data sets.

### Community Types and Long-term Stability

**Figure 6:**
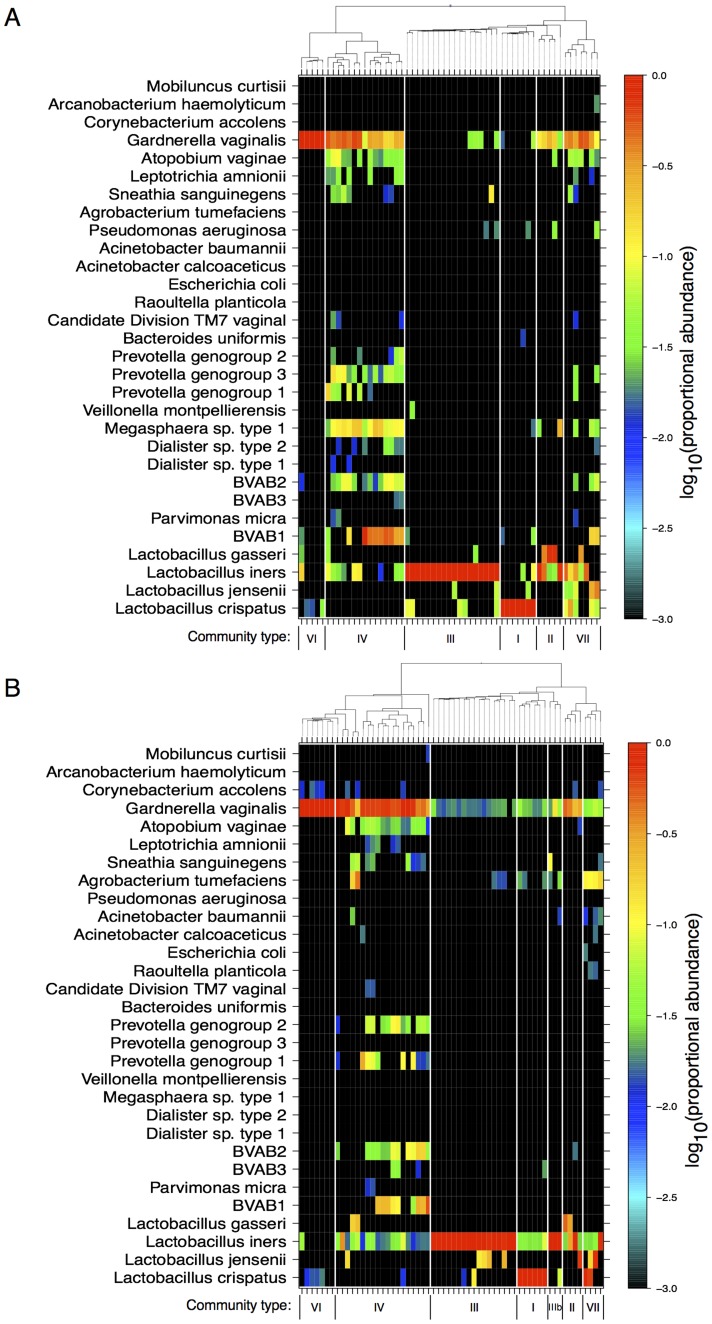
Categorical community types by squash clustering and prevalence of species. Reads from the 454 (panel A) and Illuimna (panel B) platforms were classified at the species level by pplacer and guppy, and clustered using squash clustering [25]. The figure shows the distributions of reads between species for each clinical sample as heat-maps, on a logarithmic scale, arranged according to the squash clustering. The tree produced by the clustering algorithm is shown at the top of the heat-map, with community type designations appearing below; the type names are in accord with those proposed by Ravel *et al.* [8].

To delineate community types, a combination of squash clustering [25] and scrutiny of the species-level sample classifications were performed (Fig. 6). Distinct microbial community types were observed amongst the samples, consistent with existing reports on vaginal microbiota [8]. Since pplacer provided high maximum-likelihood (>0.9) classifications of Illumina and 454 sequencing data and is compatible with a variety of useful analysis algorithms through guppy, we used the results from this software pipeline to derive categorical community types. Sequencing reads from both NGS methods organized into 6 distinct clusters (Fig. 6). Those named I–IV are analogous to the community types defined by Ravel *et al.* [8]. We did not observe their type V (dominated by *L. jensenii*), whereas our data indicated the presence of 2 additional community types, labeled VI and VII. Type VI is characterized by the almost exclusive presence of *G. vaginalis*, whereas type VII has high, approximately even proportions of *G.vaginalis* and *Lactobacillus* spp. along with low abundances of the species found in type IV. In the Illumina squash clustering (Fig. 6A), we see an additional cluster that we designated IIIb. Although this group clustered separately from III, it was also characterized by a predominance of *L. iners*.

**Figure 7:**
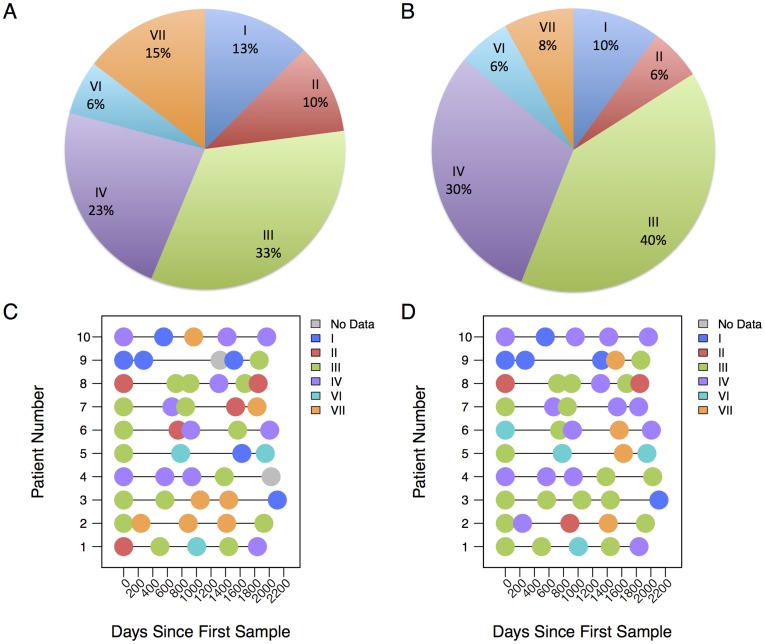
Time courses and distribution of microbiome community types. Panels A and B show the proportions of samples assigned to each community type using 454 and Illumina, respectively, for the whole study population (10 women) across all time points (5±1). Experimental replicates are excluded. Community types III and IV constitute over half of the cervical microbiome from these women. Panels C and D show the microbiome community types over time, as characterized by 454 and Illumina, respectively, when using pplacer and guppy to classify and cluster the reads.

Four of the community types detected in this study were similarly derived by Ravel *et al* [8]. In fact, the current analysis observed similar proportions of these community microbiome types across the combined sample set obtained from the population of Costa Rican women compared to those reported in the Ravel *et al.* Hispanic population [8] (Fig. 7A–B).

Examining the stability of the cervical microbiome over relatively long periods of time, (5–7 years), we observed that the categorical cervical microbiome composition appeared capable of both relative stability over years and fluctuations between a small number of defined community types (Fig. 7C–D). The 454 sequencing (Fig. 7C) and Illumina sequencing (Fig. 7D) experiments resulted in different community type assignments for some samples.

Figure S1 shows plots in the first three principal components (PCs) of species level classifications performed by the pplacer pipeline on 454 (Fig. S1B, D) and Illumina data (Fig. S1A, C). We observed fluctuation between approximately 3 clusters (Fig. S1B, D) and no consistent time-dependent trends across the 5–7 year study period for these 10 women. Points from the same community types were observed to cluster mostly together within the first three PCs (Fig. S1B, D). Movement between restricted regions of composition space indicated long-term stability of what may be thought of as stationary points in the cervical microbiome dynamical system. Figures S2A and S2B show correlation of the first two principal components with the original dimensions (species) for the 454 and Illumina data, respectively, as analyzed by pplacer. The PCA heat-maps (Fig. S2A–B) showed inverse relationships between *L. iners*, *L. crispatus* and *G. vaginalis. L. iners* and *L. crispatus* were thus somewhat mutually exclusive, as were both with *G. vaginalis*. Edge principal component analysis (EdgePCA - [25]) confirmed that the overwhelmingly dominant community transition (explaining >80% of variance in the data) is mostly accounted for by a shift between *L. iners* and *G. vaginalis* (Fig. S3).

We compared inter- and intra-subject microbiome variability using PERMANOVA [26], a non-parametric multivariate analysis of variance test that employs a permutation procedure to test the null hypothesis that there is no difference between and within subjects. A pairwise distance matrix of KR values (with KR weighting parameter, *p* = 1) between the microbiota of all samples was generated. When grouping by subject (i.e., subject was the factor), the calculated PERMANOVA *F*-test was 2.86 (p = 0.018), indicating a significant difference of microbiota within subjects compared to between subjects. Additionally, exploratory analyses of associations of cervicovaginal microbiome with HPV status (positive or negative by PCR), vaginal pH and time since last menstrual period (LMP) were performed using GEE logistic regression models with an autoregressive correlation structure to account for repeated sampling. For the Illumina-generated data, we detected an unadjusted association between cervical microbiome dichotomized according to squash clustering (Fig. 6– following the deepest bifurcation of the clustering trees) and HPV status (effect size = −1.421, p<0.005). When adjusting for all 3 variables in the model, no statistically significant associations were found for data generated by either sequencing platform and the relatively small sample number limited our statistical power.

## Discussion

We undertook a comparison of the classifications of cervical microbiota produced by 3 different sequencing methods in combination with 3 analysis pipelines based on distinctly different classification algorithms. In addition, sampling subjects over the course of 5–7 years allowed us to assess long-term stability of the cervical microbiome in the early-sexual life of women. Our results indicate that the BLAST-like usearch algorithm and the maximum-likelihood phylogenetic placement algorithm, pplacer, in combination with the guppy classifier, generated similar classifications for Roche 454 and Illumina HiSeq2000 reads. The Naïve Bayes RDP classifier produced similar results to those of usearch and pplacer on the longer 454 reads, but differed significantly when classifying the shorter Illumina reads [23].

Squash clustering and examination of species associated with the clusters demonstrated the presence of distinct bacterial community types within the cervix (Fig. 6). Their broad agreement with those found in the vaginal microbiome literature [8], [21] indicates that despite reported differences between the communities present at different sites within the reproductive tracts of individual women [9], overall compositions from different women, different analyses and different laboratories generate remarkably similar overall patterns. In addition, it further validates the approach of characterizing cervicovaginal bacterial communities into a small number of discrete states, or “community types”.

Comparing the phylogenetic read placement of trimmed 454 data (V6) to full-length 454 data (V6–V9) produced very similar placements (Table 1) and suggests that discrepancies between the Illumina and 454 data are the result of differential PCR amplification bias between the two primer sets. Nevertheless, based on the high average level of reproducibility observed (Fig. S4), it would seem that the degree of PCR bias is consistent for a given set of PCR primers and target region. This result becomes important for future studies of any microbial environment using 16S rRNA and massively parallel short-read sequencing. It further suggests that PCR-independent library preparation, where possible, should reduce bias and improve accuracy. In addition, obtaining a complete and accurate representation of microbiome composition may require assaying multiple genes or gene regions.

The methodological comparisons and the processes involved in producing each of the sets of data served to illuminate pros and cons of the different methodologies for different sets of circumstances. Cloning and Sanger sequencing yielded a tractable set of data and was sufficient for low-depth analysis of a microbiome, which in many cases would serve to accurately detect the abundances of the predominant microbes (see Figs. 3 and 4). In fact, a rarefaction analysis indicated that somewhere between 100 and 500 random reads should be sufficient to accurately characterize the cervicovaginal microbiome diversity (see Fig. S5). This fact should guide future studies to reduce wasted depth of sequencing. Nevertheless, cloning and sequencing suffers from being labor- and resource-intensive and a cost-per base that is several orders of magnitude higher than for the NGS platforms. In general, the Illumina platform provides a greater number of reads, similar ability to distinguish between bacteria (often down to the species level) and for a fraction of the cost of 454 pyrosequencing. The usearch pipeline is useful for searching against a custom database, as a simple unaligned FASTA format file of reference sequences is all that is required. With appropriate parameters it appears that the classifications produced are very similar to those produced by pplacer for both read lengths. A drawback of usearch potentially occurs when two sequences in the database match a read equally well; since by default, it yields only the first best match. Reporting all best matches by manipulating the parameters and using some additional post-classification software would, however, allow one to overcome the problem to some extent. A user-friendly software pipeline for microbiome classification now exists for usearch, called “otupipe”. RDP Classifier and pplacer have stringent reference database requirements and it is often far from trivial to produce high-quality custom databases for use with these pieces of software. Moreover, it has been shown that the reference database significantly affects the quality of classification results [15] and therefore, it is a step deserving time and effort. In addition, well-curated and frequently updated databases are available for many common applications. If a database is available, RDP Classifier produces classifications with the least amount of additional pre- or post-processing and performs rapidly and consistently with other methods where reads are >250 bp [22]. It should be noted that recent advances in Illumina technology (2×150 bp) pushes maximum read lengths towards RDP Classifier’s high-accuracy range. Pplacer, though requiring a number of pre- and post- processing steps to produce classification tables (see Fig. 2), offers many sophisticated analysis options for use with its output, as well as the placement of all reads on a reference phylogenetic tree, based on high performance algorithms and rigorous statistical methods [19], [24], [25].

Analysis of the multiple and long-term sampling of microbiota in these cervical specimens showed fluctuation within a narrow region of composition space and supports the hypothesis that a small number of stationary points exist between which the cervical microbiome can fluctuate following sources of perturbation (Figs. 6, 7C–D and S2). No substantial divergence from this behavior was observed over the 5–7 year period, although our sample size was relatively small and additional studies are needed. Our samples were selected from young Costa Rican women, but taken with the rest of the current cervicovaginal microbiome literature, suggest that the hypothesis is likely to apply for all sexually active women [27]. Furthermore, the observed dependence of microbiome composition and variability upon subjects (the PERMANOVA analysis) highlights the importance of longitudinal data in microbiome studies of the cervix. We believe the emphasis now lies on determining the association of these characterized microbiome states (i.e., community types) with the factors that drive microbiome transitions and with pathological outcomes in long-term prospective studies (for recent review see [3]).

## Methods

### Sample Collection and Study Design

Cervical samples were obtained from a large population-based cohort study (10,049 women) conducted in Guanacaste, Costa Rica, previously described [17], [28], [29]. For the current study, 10 women who recently initiated sexual activity and had yearly samples available were randomly selected for this analysis [29]. Samples were obtained during a pelvic exam by specially trained nurses using a nonlubricated sterile speculum. The cervical specimens for the microbiome analyses were initially collected for HPV DNA testing using a Dacron swab (Digene, Gaithersburg, MD; now part of Qiagen, Hilden, Germany) that was swabbed over the ectocervix and rotated in the endocervical canal and placed into either ViraPap DNA transport medium or sample transport medium (STM) (Digene), as described [16]. The total observation period for each woman spans 5–7 years. Costa Rican and National Cancer Institute of the United States institutional review boards and the Committee on Clinical Investigation at the Albert Einstein College of Medicine approved all study protocols. All participants signed an informed consent form.

### DNA Extraction and Amplification

Briefly, an aliquot of each cervical sample was incubated with a proteinase K and sodium laureth-12 sulfate solution and DNA was then precipitated in a 0.825 M ammonium acetate/ethanol (AAE) solution, pelleted by centrifugation and resuspended in TE, as described previously [17], [28]. Samples for analyses by cloning and Sanger sequencing and for sequencing by Roche 454 were PCR amplified using primers to an approximately 525 bp region spanning the V6–V9 region (target primer sequences were kindly provided by Julie Segre [30]). Samples for sequencing by Illumina were PCR amplified using primers to an approximately 145 bp region spanning the V6 region (target primer sequences obtained from [31]). For all samples, a unique DNA barcode was introduced to the PCR amplicons by the PCR primers. Barcodes used for Roche 454 sequencing were 10 bp in length and were appended to the 3′ terminal end of the amplicon, whereas barcodes used for Illumina sequencing were 8 bp Hamming barcodes [32] and different codes were appended to 3′ and 5′ terminal ends of PCR amplicons to allow separation of forward and reverse sequences. Figure 1 shows primer design and target primer sequences for both sets of primers. Successful amplification of the predicted fragment size was confirmed and amplicon concentration estimated by relative band brightness against a control using gel electrophoresis [33].

### DNA Sequencing

The three sequencing techniques used in this study were (1) cloning + dideoxy sequencing (Sanger sequencing on an ABI 3730 DNA Analyzer), (2) direct pyrosequencing (Roche 454 GS-FLX) and, (3) short-read sequencing-by-synthesis (Illumina HiSeq2000). Short reads from all three platforms were deposited in the NCBI Sequence Read Archive (SRA - http://www.ncbi.nlm.nih.gov/sra) under SRA052206.

### Cloning and Sanger Sequencing

Thirty of the 50 PCR amplicons of the 16S V6–V9 region were cloned into E. coli using the One Shot® MAX Efficiency® DH5α™-T1^R^ TOPO-TA cloning kit (Invitrogen Corporation, Carlsbad, CA, USA). Between 50 and 94 colonies per sample were selected and sequenced by Genewiz (South Plainfield, NJ, USA), of which 30–81 were successfully sequenced. The first, last and middle time point for each of the 10 women were used for this analysis.

### Massively Parallel Sequencing

Prior to sending samples for NGS, barcoded PCR products from all clinical samples were pooled at approximately equal molar DNA concentrations and run on a preparative agarose gel. The correct sized band was excised, the DNA was electroeluted, precipitated in ethanol and resuspended in TE buffer as previously described [33]. One aliquot of pooled, purified, barcoded DNA amplicons was sequenced on a Genome Sequencer FLX System (Roche 454 Life Sciences, Branford, CT, USA), with long-read Titanium chemistry, by SAIC-Frederick, Inc., National Cancer Institute (Frederick, MD, USA), another similarly prepared pool of amplicons was sequenced on an Illumina HiSeq2000 (Illumina Inc., San Diego, CA, USA) by the Epigenomics and Genomics Core Facility, Albert Einstein College of Medicine (Bronx, NY, USA) using single-end reads.

### Software

To process the Sanger, Roche 454 and Illumina reads that allowed comparison between classification results produced by the 3^rd^-party taxonomy software used (usearch [18], RDP Classifier [14], and pplacer [19]), a number of python v.2.7, shell and sqlite3 scripts were developed in-house (Fig. 2). These have been bundled and are available for download as a python package called “mubiomics” on sourceforge (http://www.sourceforge.net/projects/mubiomics) and github (http://www.github.com/benjsmith/mubiomics). The functions of the quality control and demultiplexing software were inspired by QIIME [34]; but, at the time of study it was necessary to develop in-house scripts to process the millions of short Illumina reads, since these were not handled by the QIIME pipeline. Later releases of QIIME do handle Illuimna reads. Preliminary 454 data was processed with QIIME, however all data analyzed in this report was processed with the in-house software, for consistency.

In addition to usearch, RDP Classifier and pplacer, other available software was used to facilitate the analysis and comparison of data. Guppy (http://matsen.fhcrc.org/pplacer/) was used to analyze the placements produced by pplacer, produce phylogenetic trees with branch line widths proportional to the number of assignments, perform squash clustering, edge principle component analysis (EdgePCA), calculation of Kantorovich-Rubinstein (KR) distances (equivalent to the weighted UniFrac distance [24], [35]), and production of phylogenetic trees for visualizing pairwise KR distance. Archaeopteryx [36] was used for visualizing trees in XML format produced by guppy, and FigTree v1.3.1 (http://tree.bio.ed.ac.uk/software/figtree) was used to visualize trees in Newick format produced by guppy’s squash clustering program.

### Bioinformatics

Several bioinformatics pipelines, consisting of a combination of publicly available software and those developed in-house to handle the different data and formats for this study were used for analyzing the nucleotide data output by the various sequencing methods. Figure 2 shows a flow chart of the pipelines and associated software. These include, as shown in panel 3, (A) an enhanced BLAST-like algorithm (usearch [18]), (B) a naïve Bayes classifier (RDP classifier [14]), and (C) a phylogenetic placement algorithm (pplacer [19]). Parameter settings can be obtained from the authors upon request. Briefly, from the Sanger sequencing we obtained ABI files containing both sequence and quality data; from the Roche sequencer, we obtained a pair of FASTA and QUAL files containing sequence and quality data, respectively; and from the Illumina sequencer, we obtained a FASTQ file containing both sequence and quality data. All pipelines then performed the following steps as shown in Figure 2: Quality control filtering (panel 1); Read demultiplexing (i.e., assigning original sample identities to reads according to DNA barcode – panel 2); Read identification (i.e., assigning a bacterial identity to a sequence – panel 3); Sample composition reporting (i.e., consolidating results from individual reads into an identical table for each pipeline – panel 4).

To produce the classification tables from which Figure 3 was generated (panel 4 in the flowchart of Fig. 2), compositions were summarized by proportion at the genus level. All sequences that were assigned a taxonomic identifier at levels above this, e.g. family, were not included in the analyses, whereas all sequences that were assigned at levels below this, e.g. species, were grouped by their corresponding genera. In some cases (e.g., the BVAB strains), reads were identified as originating from sequences in the database with incomplete taxonomic information (i.e., although classified at the genus level, an official genus-level taxonomic identifier wasn’t present in the database). In such cases, an appropriate identifying name was assigned at the genus level (e.g., the species name for *Parvimonas micra*, the strain names for *BVAB1*, *2* and *3*, and the order name for the genus below *Clostridiales*), this is reflected in the figures by the presence of an asterisk. The set of genera that constitute the rows in the heat-maps of Figure 3 is a union across all 9 proportional compositional tables. That is, each genus shown in Figure 3 appeared in at least 1 sample in at least 1 of the 9 methodological configurations, with sufficient reads to survive the filtering (i.e., each genus constitutes ≥1% of a sample’s community).

For all analysis methods, read classification was performed against the vaginal microbiome 16S rRNA database ([19] - bundled with pplacer at the time of writing). In order to train this database for use with RDP Classifier, a custom python script (“taxtastic2rdp.py”) was written to convert the available files to requisite input files for the RDP Classifier training software. This can be found in the mubiomics package (see software section above).

### Statistical and Comparative Analyses

All plotting and statistical comparisons were performed in R v2.12.2 using a script developed in-house (available upon request). Difference matrices used to compare sequencing methods (Fig. 4A) were calculated by subtracting one proportional composition matrix from another. To compare each combination of sequencing technology and analytical pipeline, columns in which data were present were averaged across samples and across genera. To assess the degree of reclassification for each genus (Fig. 4B), counts for each genus were summed over samples and divided by the total number of counts in the classification table, to produce a proportion of reads assigned to each genus. This was done for each methodological configuration and the distributions visualized as boxplots. Shannon diversity indices (Fig. 5) were calculated in R using the standard formula for Shannon entropy [37].

Squash clustering [25] (trees at top of Fig. 6A–B), which is based on KR distance [24] (a generalized version of the UniFrac distance [35]), between samples was performed using the *guppy squash* subcommand with *p* = 1 [24], [25]. As the value of parameter *p* is increased in the KR distance metric, the weighting of the distance value shifts from emphasizing the amount of phylogenetic distance travelled to the number of reads repositioned [24].

To evaluate the effects of PCR bias, 454 reads longer than 120 bp were truncated and processed according to the pplacer pipeline (Fig. 2, box C). The read placements from all 60 samples for the full-length 454 reads, the truncated 454 reads and the Illumina reads were pooled and the *guppy kr* subcommand [19] was used to calculate the tree-length normalized KR distance (*Z_p_*) between all pair-wise combinations of the three data sets. Since many more reads were generated in the Illumina run, we set *p* = 1 to give more weight to the distance of transport.

Principal component analysis (Figs. S1, S2) was performed in R v.2.12.2 using the *prcomp* function. Figure S3 was calculated using the *guppy pca* subcommand with the default parameters and visualized using Archaeopteryx (described under “Software”, above). For Figure S4 (reproducibility analysis), all pairwise KR-distances between samples were calculated using the *guppy kr* subcommand with *p* = 1 [24], [25] and normalizing with respect to the diameter of the reference tree. Rarefaction curves (Fig. S5) were calculated using the *rarecurve* function in R’s *vegan* package. Input data were species-level classification tables produced by running the *pplacer* pipeline on reads generated by the 3 sequencing platforms. The PERMANOVA analysis [26], with subject as the grouping factor, was performed using the *betadisper* and *permutest* functions in R’s *vegan* package.

Explorations of associations amongst the microbiome, HPV status (positive or negative by PCR) [29], vaginal pH [5], and days since last menstrual period (LMP) were calculated via a Logistic Regression GEE model. The auto-regressive correlation structure (AR1) was used due to the longitudinal sampling of the data. The microbiome was dichotimized based on the squash clustering, cut at the first (deepest) bifurcation of the tree. This corresponded to community types IV+VI vs. everything else (see Fig. 6). HPV was a categorical variable based on status while pH and LMP were considered interval data. Analyses were performed using SAS Version 9.2 (SAS Institute, Cary, NC).

### Reproducibility

Experimental reproducibility was assessed by repeat testing of 10 of the 50 samples. Figures S4A and S4B show a high correlation between the proportions of species produced by original samples and their repeat measurements (R^2^ = 0.995 for 454 (Fig. S4A) and Illumina (Fig. S4B)). Figure S4C shows the results of a KR dissimilarity analysis, performed on assignments generated by pplacer on the repeat measurements of truncated 454 reads, full-length 454 reads, and Illumina reads. Both methods also exhibited a similar level of reproducibility by this analysis (Illumina proportional normalized KR distance (Z_1_): median = 0.020, mean = 0.066, IQR = 0.087; 454 (truncated and full length) Z_1_: median = 0.036, mean = 0.052, IQR = 0.029). Since the normalized KR distance is presented here as a proportion of the maximum normalized KR distance observed between any two samples within a set of experimental conditions, Figure S4C can be interpreted as showing the amount of experimental error relative to the largest differences observed in the experiment. In this analysis (Figure S4C), the pairwise KR distances between samples and their repeats (calculated by guppy, as described above), were normalized with respect to the diameter of the reference tree and divided by the largest observed KR distance amongst all samples for the relevant sequencing method.

## Supporting Information

Figure S1 **Principal component analysis of cervical microbiota.** Panels A and C contain histograms showing the proportion of variance associated with the first 10 principal components (PCs) of species-level composition matrices (classified by pplacer and guppy) for 454 and Illumina sequencing runs, respectively. Panels B and D contain PCA plots showing the degree of correlation of samples with principal components 1, 2 and 3 (PC1, PC2, and PC3) for 454 and Illumina, respectively. Points were colored according to the categorical microbiome community type to which they belonged as indicated by the legend in the box above the plots. The dropdown lines indicate the position of PC3 on the PC1 and PC2 two-dimensional plane.
(TIFF)

Figure S2 **Correlation of first three principal component dimensions with categorical species types.** Principal components analysis finds linear combinations of the original dimensions of a set of high-dimensional data to form new dimensions that explain decreasing proportions of the variance within the data. The heat-maps in panels A and B show the correlation of the first three transformed dimensions- PC1, PC2, and PC3 with the original dimensions (species) for 454 and Illumina data, respectively, as classified by pplacer. This figure can be viewed in conjunction with Fig. S1 in order to understand the composition of PCs 1, 2, and 3.
(TIFF)

Figure S3 **Edge principal component analysis.** The figure shows the first principal component of an EgdePCA [25] performed using the Illumina sequence data that was phylogenetically sorted by pplacer. Branches are fattened according to the quantity of reads that comprise the 1^st^ edge principal component and colored according to whether reads moved towards (blue) or away (red) from the root.
(TIFF)

Figure S4 **Reproducibility analysis.** Roche 454 (panel A) and Illumina (panel B) scatter plots show the proportion of each species present in the original samples and their repeats, using species-level composition tables produced from pplacer classifications. Correlations between the data are shown at the top of the plots (R^2^ values). The sample names displayed in the legend reflect patient by number and sampling time point by the succeeding letter and correspond to those in Figs. 3 and 6. Panel C, KR dissimilarity boxplot shows the median, interquartile range and spread of tree-length normalized Kantorovich-Rubinstein (KR) distances between samples and their repeat measurements, plotted as proportions of the largest normalized KR distance observed between any samples.
(TIFF)

Figure S5 **Species rarefaction curves for each sequencing method.** Panels A, B, and C show how species richness in a sample depends on the number of 16S amplicons sequenced by Sanger, 454 and Illumina platforms, respectively. A maximum of 12 (panel A), 13 (panel B), and 10 (panel C) species were observed in any one sample analyzed by the Sanger, 454 and Illumina platforms, respectively. The predicted diversity in a number of samples wasn’t completely captured by sequencing <100 molecules (Panel A), but it was nearly always obtained when sequencing >500 molecules (panels B and C).
(TIFF)
