# Supplementary figures and images for: The Cervical Microbiome over 7 Years and a Comparison of Methodologies for Its Characterization
Source: PLoS One. 2012 Jul 9;7(7):e40425. doi: 10.1371/journal.pone.0040425 (PMC3392218; doi:10.1371/journal.pone.0040425)

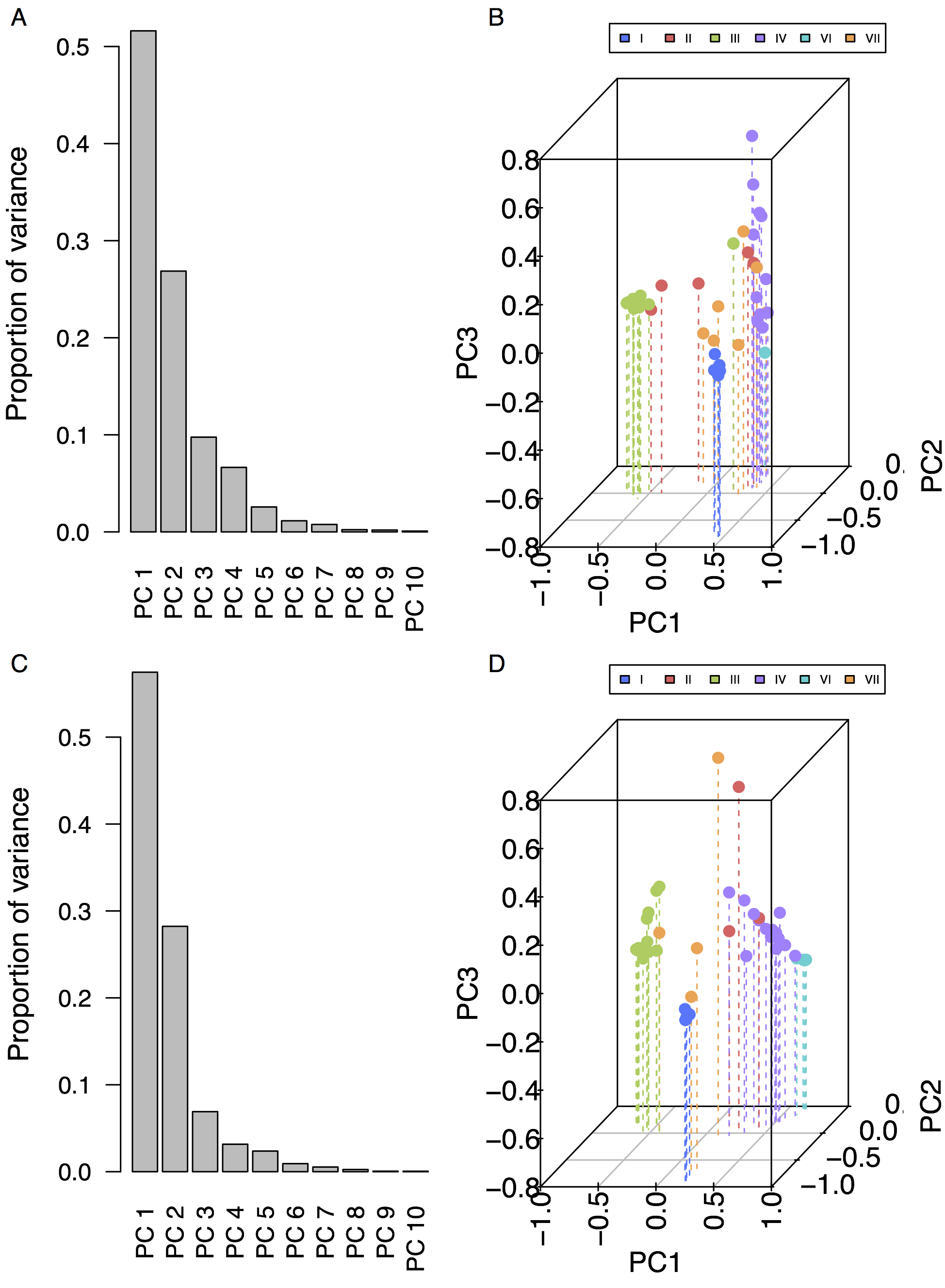

Supplement: Figure S1 — Principal component analysis of cervical microbiota. Panels A and C contain histograms showing the proportion of variance associated with the first 10 principal components (PCs) of species-level composition matrices (classified by pplacer and guppy) for 454 and Illumina sequencing runs, respectively. Panels B and D contain PCA plots showing the degree of correlation of samples with principal components 1, 2 and 3 (PC1, PC2, and PC3) for 454 and Illumina, respectively. Points were colored according to the categorical microbiome community type to which they belonged as indicated by the legend in the box above the plots. The dropdown lines indicate the position of PC3 on the PC1 and PC2 two-dimensional plane. (TIFF) [file pone.0040425.s001.tiff]

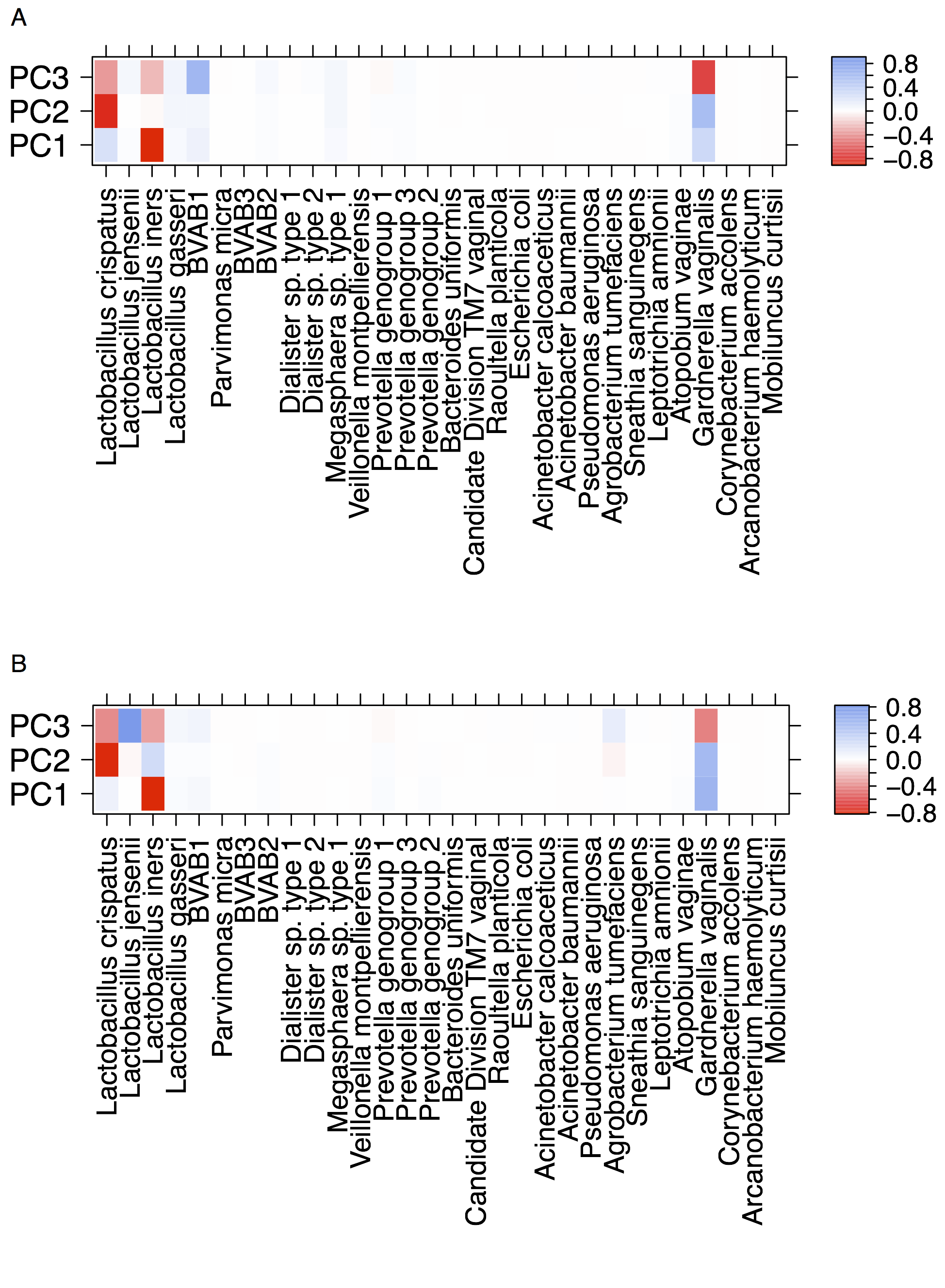

Supplement: Figure S2 — Correlation of first three principal component dimensions with categorical species types. Principal components analysis finds linear combinations of the original dimensions of a set of high-dimensional data to form new dimensions that explain decreasing proportions of the variance within the data. The heat-maps in panels A and B show the correlation of the first three transformed dimensions- PC1, PC2, and PC3 with the original dimensions (species) for 454 and Illumina data, respectively, as classified by pplacer. This figure can be viewed in conjunction with Fig. S1 in order to understand the composition of PCs 1, 2, and 3. (TIFF) [file pone.0040425.s002.tiff]

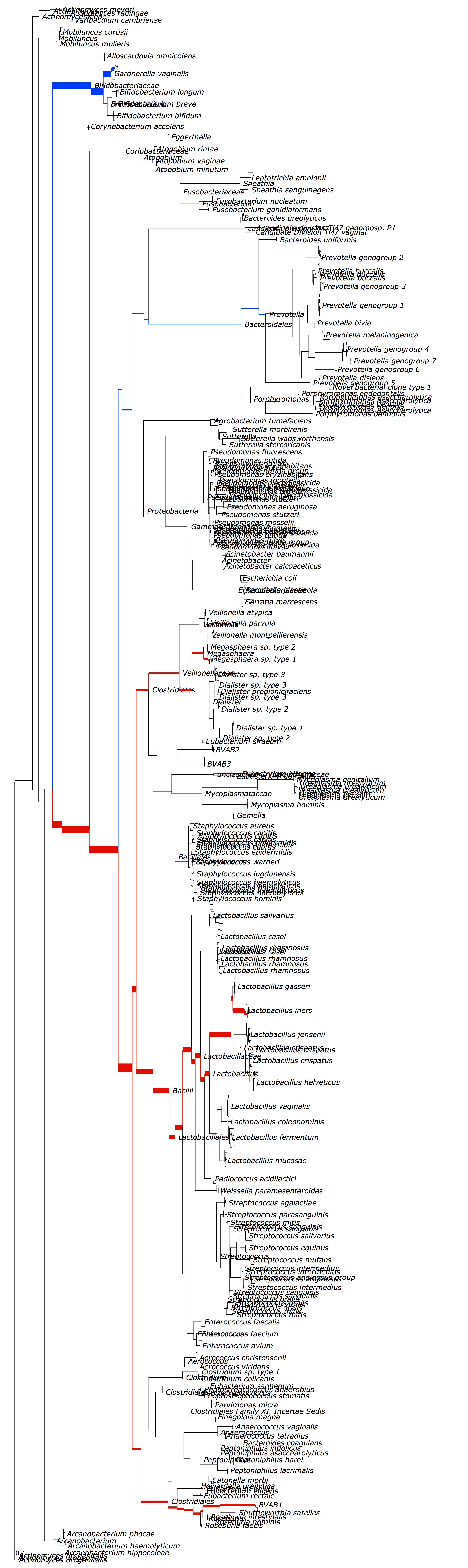

Supplement: Figure S3 — Edge principal component analysis. The figure shows the first principal component of an EgdePCA [25] performed using the Illumina sequence data that was phylogenetically sorted by pplacer. Branches are fattened according to the quantity of reads that comprise the 1st edge principal component and colored according to whether reads moved towards (blue) or away (red) from the root. (TIFF) [file pone.0040425.s003.tiff]

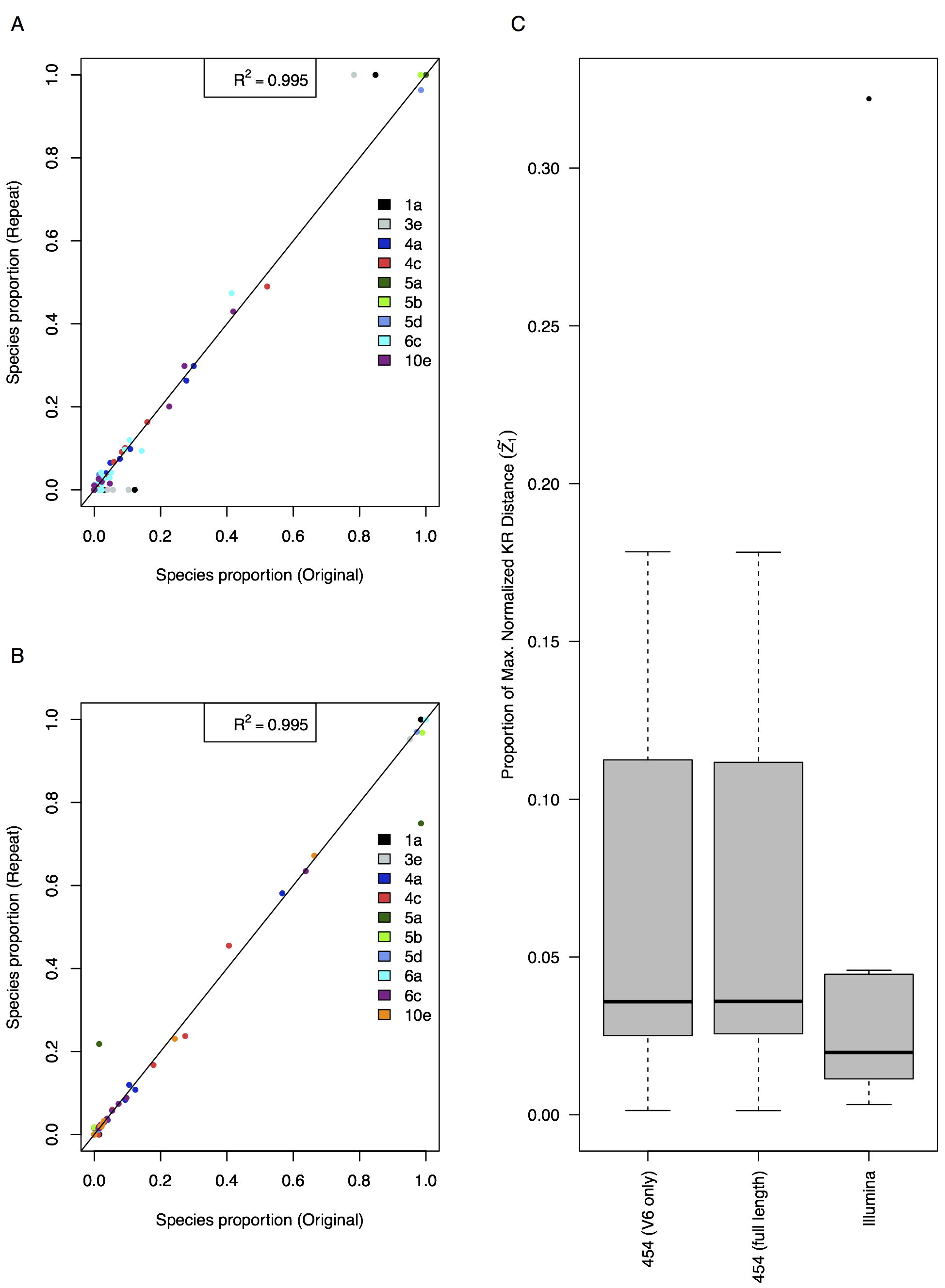

Supplement: Figure S4 — Reproducibility analysis. Roche 454 (panel A) and Illumina (panel B) scatter plots show the proportion of each species present in the original samples and their repeats, using species-level composition tables produced from pplacer classifications. Correlations between the data are shown at the top of the plots (R2 values). The sample names displayed in the legend reflect patient by number and sampling time point by the succeeding letter and correspond to those in Figs. 3 and 6. Panel C, KR dissimilarity boxplot shows the median, interquartile range and spread of tree-length normalized Kantorovich-Rubinstein (KR) distances between samples and their repeat measurements, plotted as proportions of the largest normalized KR distance observed between any samples. (TIFF) [file pone.0040425.s004.tiff]

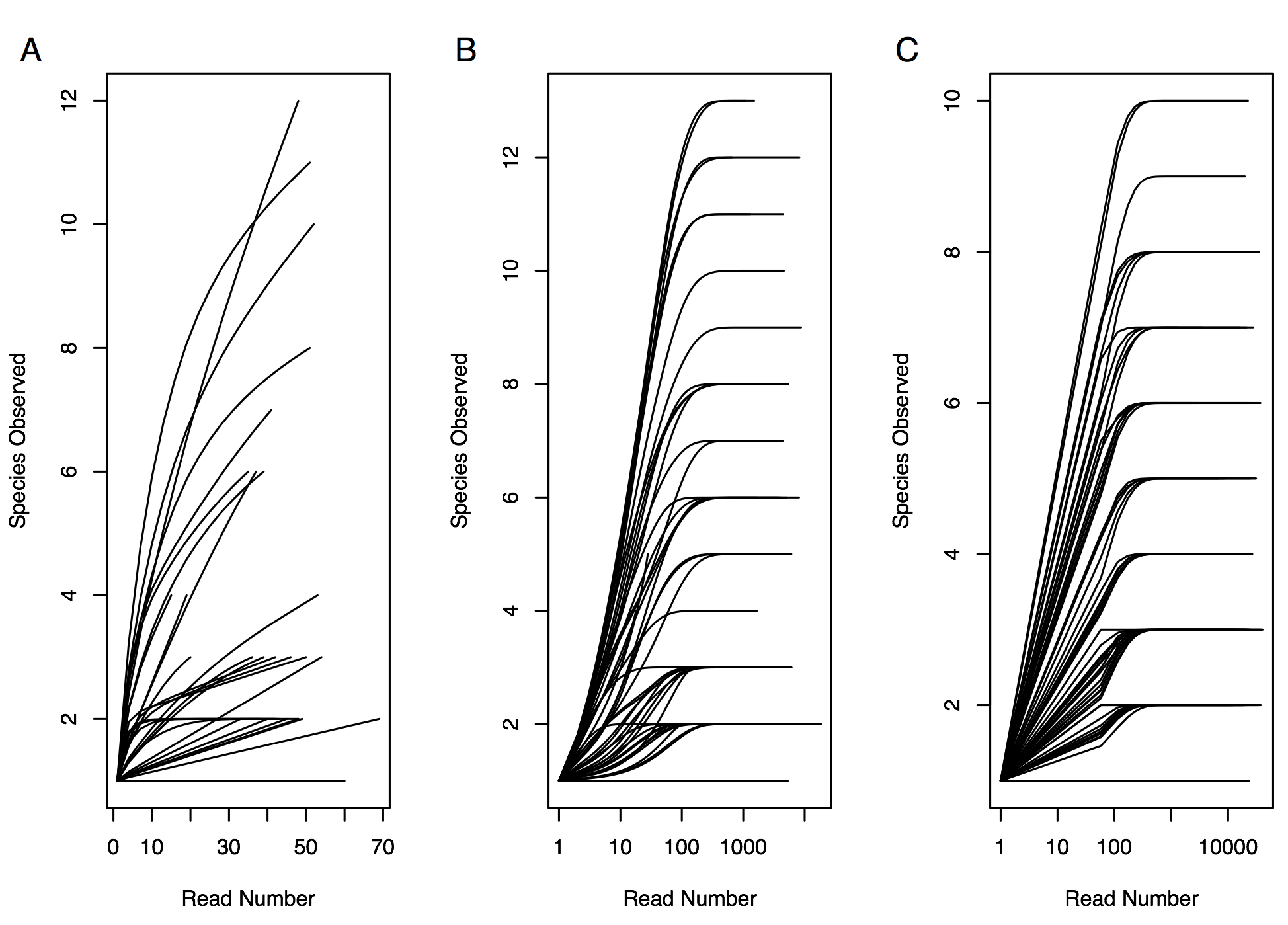

Supplement: Figure S5 — Species rarefaction curves for each sequencing method. Panels A, B, and C show how species richness in a sample depends on the number of 16S amplicons sequenced by Sanger, 454 and Illumina platforms, respectively. A maximum of 12 (panel A), 13 (panel B), and 10 (panel C) species were observed in any one sample analyzed by the Sanger, 454 and Illumina platforms, respectively. The predicted diversity in a number of samples wasn’t completely captured by sequencing <100 molecules (Panel A), but it was nearly always obtained when sequencing >500 molecules (panels B and C). (TIFF) [file pone.0040425.s005.tiff]
